# Erythematous plaque in the axilla

**DOI:** 10.1016/j.jdcr.2024.07.005

**Published:** 2024-07-26

**Authors:** Rachel Fayne, Ji Won Ahn, Kelly L. Harms

**Affiliations:** a Department of Dermatology, University of Michigan, Ann Arbor, Michigan; b Department of Dermatology, University of Pittsburgh Medical Center, Pittsburgh, Pennsylvania

**Keywords:** axillary extramammary Paget’s disease, intertriginous rash, Paget’s disease

## Case presentation

**Fig 1:**
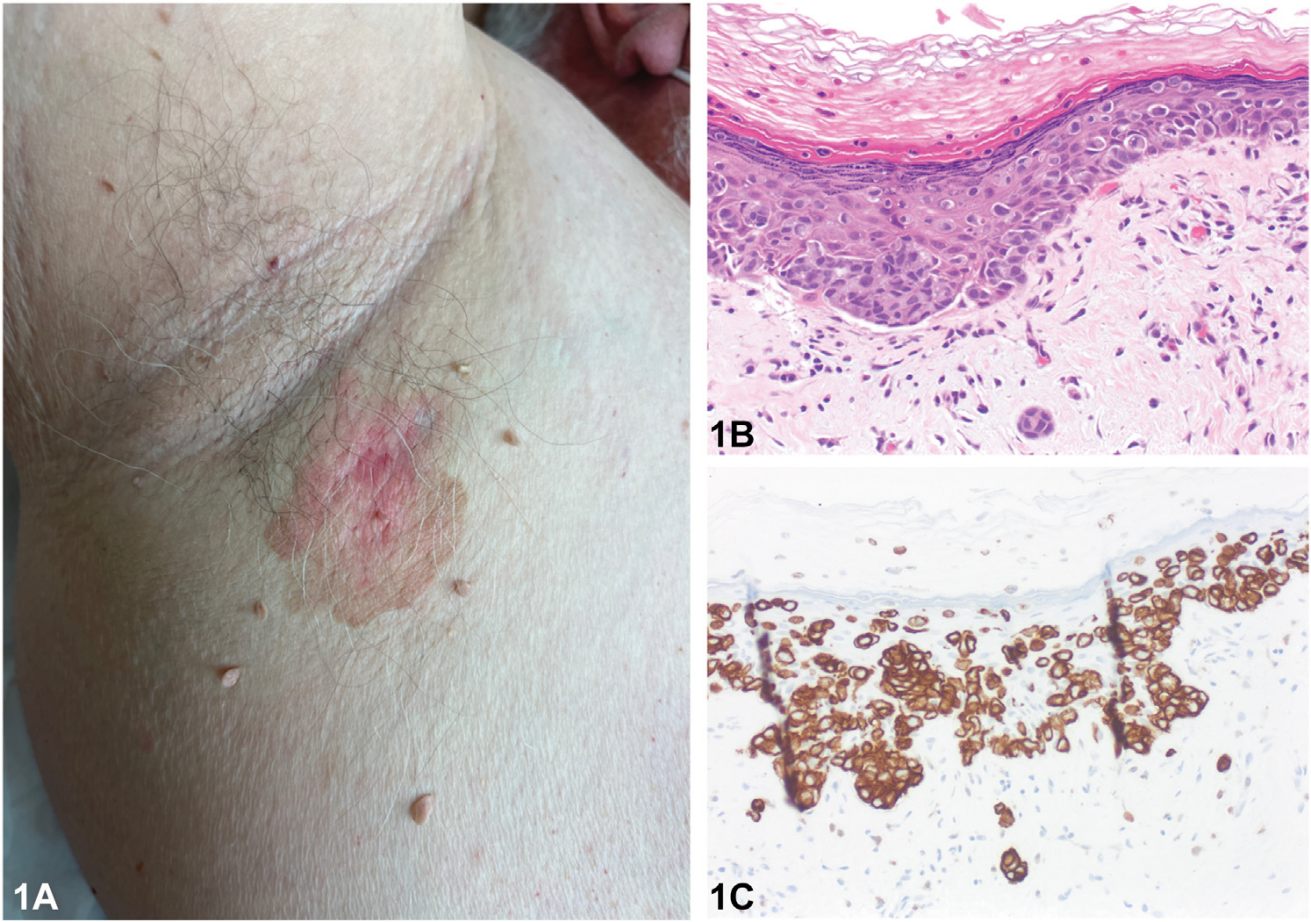

A Caucasian man in his 70s presented with a history of an itchy rash in his right axilla. Physical examination revealed a reddish-brown, well-demarcated plaque in the right axilla (Fig 1, *A*). A biopsy was performed after failure to treat empirically. Biopsy demonstrated intraepidermal carcinoma with areas of focal dermal invasion (Fig 1, *B* and *C*). Immunophenotyping demonstrated positive staining for cytokeratin 7 (Fig 1, *C*), CAM5.2 and CEA staining in numerous epithelioid cells scattered throughout the epidermis in a buckshot pattern. CK5/6, p40, and SOX10 were negative.

**Question 1: What is the most likely diagnosis?**

A. Erythrasma
B. Irritant contact dermatitis (ICD)
C. Extramammary Paget’s disease (EMPD)
D. Candida intertrigo
E. Inverse psoriasis

**Answers:**

A. Erythrasma – Incorrect. Erythrasma is usually characterized by hyperpigmented or erythematous patches in intertriginous areas. Wood’s lamp exam may show coral red fluorescence. Biopsy would demonstrate small filamentous organisms, Corynebacterium minutissimum, in the stratum corneum.
B. Irritant contact dermatitis (ICD) – Incorrect. ICD will frequently demonstrate vesicles, serous crusting, scale or erosions in acute disease, or lichenification in more chronic disease. Biopsy would reveal a spongiotic dermatitis with dyskeratosis.
C. Extramammary Paget’s disease (EMPD) – Correct. EMPD is a rare cutaneous malignancy that most commonly presents on apocrine gland-bearing skin, most often the anogenital region.^1^ Less frequently, EMPD affects other areas with apocrine glands, including the axilla. To date, including our case, approximately 50 cases of axillary EMPD have been reported, mainly in Chinese and Japanese patients.^1^ This case serves as an important reminder that EMPD is not exclusively a disease of the anogenital region, and to consider EMPD in axillary rashes that do not respond to first-line therapies for common intertriginous rashes like intertrigo, ICD, inverse psoriasis, or erythrasma. Biopsy result described is consistent with EMPD.
D. Candida intertrigo – Incorrect. Intertrigo typically shows maceration, erosions, or fissuring and is due to friction in skin folds. In candida intertrigo, satellite lesions may be present, and biopsy would reveal pseudohyphal fungal forms.
E. Inverse psoriasis – Incorrect. Inverse psoriasis presents as reddish-brown shiny thin plaques in areas of skinfolds such as axilla, groin, and gluteal cleft. Biopsy reveals features specific for psoriasis such as confluent parakeratosis, psoriasiform hyperplasia, and intracorneal neutrophils.

**Question 2: What internal malignancy can be associated with this diagnosis?**

A. Follicular thyroid carcinoma
B. Myeloid blood dyscrasia
C. Medullary thyroid carcinoma
D. Breast cancer
E. Medulloblastoma

**Answers:**

A. Follicular thyroid carcinoma – Incorrect. Follicular thyroid carcinoma is classically associated with Cowden syndrome.
B. Myeloid blood dyscrasia – Incorrect. Myeloid blood dyscrasia is associated with pyoderma gangrenosum.
C. Medullary thyroid carcinoma – Incorrect. Medullary thyroid carcinoma is classically associated with multiple endocrine neoplasia or Birt-Hogg-Dubé.
D. Breast cancer – Correct. It is recommended that cancer screening should be performed based upon the location of the EMPD. With disease in the axilla, the patient was screened for breast cancer with a mammogram. Anoscopy or colonoscopy may be utilized to rule out a suspected primary anal, rectal, or colon cancer in cases of perianal EMPD. Urine cytology and may be utilized to rule out a suspected primary bladder or urothelial cancer in cases of vulvar or penile EMPD.^2^
E. Medulloblastoma – Incorrect. Medulloblastoma is classically associated with Gorlin syndrome. Germline mutation *PTCH1* is the most common mutation.

**Question 3: Further work up showed that this was localized to the skin without underlying malignancy. Which of the following is the most appropriate treatment recommendation?**

A. Radiotherapy
B. 5-Fluorouracil cream
C. Excision (with Mohs micrographic surgery, staged excision with complete margin assessment, or wide local excision)
D. Imiquimod cream
E. Chemotherapy

**Answers:**

A. Radiotherapy – Incorrect. In this case with dermal invasion, definitive surgical treatment is favored. Definitive radiotherapy may be considered in patients who are not surgical candidates based upon disease extent or medical comorbidities.^2^
B. 5-Fluorouracil cream – Incorrect. In this case with dermal invasion, definitive surgical treatment is favored. Data is limited regarding utilization of 5-fluorouracil for EMPD.^2^
C. Excision (with Mohs micrographic surgery, staged excision with complete margin assessment, or wide local excision) – Correct. EMPD is treated with wide local excision, staged excision with complete margin assessment, or Mohs micrographic surgery.^2^, ^3^, ^4^ Recurrence is possible even with negative surgical margins as EMPD can be noncontiguous. Reports of recurrence with axillary EMPD are low, from approximately 0% to 9%.^2^^,^^4^ The literature does not definitively support or recommend against sentinel lymph node biopsy; however, clinician lymph node examination should be performed during evaluation and subsequent work-up initiated if palpable lymphadenopathy is identified. In our case, the patient underwent wide local excision with 1.5 cm margins and sentinel lymph node biopsy, which yielded negative surgical margins and 3 lymph nodes negative for carcinoma. Eight months after initial diagnosis, the patient continues to do well without clinical evidence of recurrence.
D. Imiquimod cream – Incorrect. In this case with dermal invasion, definitive surgical treatment is favored. Recently published clinical practice guidelines recommend that imiquimod cream may be considered for patients who are not surgical candidates or as an adjuvant approach for positive surgical margins when additional surgery is not feasible.^2^ However, a recent systematic review analyzed 24 studies and demonstrated a 48% complete response rate using imiquimod treatment.^5^
E. Chemotherapy – Incorrect. In this case with dermal invasion, definitive surgical treatment is favored. Chemotherapy would be indicated in the metastatic setting.^2^

## Conflicts of interest

None disclosed.
